# Can location cues facilitate attentional suppression?

**DOI:** 10.1177/17470218251357942

**Published:** 2025-07-03

**Authors:** Daniel Poole, Jim Grange, Elizabeth Milne

**Affiliations:** 1 The University of Sheffield, Sheffield, UK; 2 Keele University, Keele, UK

**Keywords:** Attention, suppression, cueing, expectation, distractor

## Abstract

The spatial cueing paradigm has illustrated that location cues result in attentional enhancement of target stimuli. However, evidence is mixed on whether proactive attentional suppression can be cued similarly. In this registered report, we used a hybrid flanker-visual search-spatial cueing paradigm in which participants were presented with informative or non-informative cues regarding the upcoming location of a target feature matching a distractor in the search array. We aimed to replicate and extend a previous study which found evidence that cues support attentional suppression. We repeated the experiment with informative and non-informative cue conditions blocked (Experiment 2) and with possible target and distractor locations separated (Experiment 3). Across all three experiments (total *n* = 554), we did not observe any evidence of cueing-enhanced attentional suppression. In Experiments 1 and 3, participant responses were slightly slower in the informative cue condition, suggesting that the cue itself captured attention when the cue type was interleaved and thus unpredictable trial-to-trial. Surprisingly, post-experiment assessment of distractor learning suggested participants had not learnt the association between cue and distractor location in any experiment. These findings do not support spatial cue-enhanced attentional suppression.

## Introduction

Selective attention refers to the prioritised processing of target stimuli while suppressing others in the presence of multiple stimuli. The neurocognitive mechanisms involved in selectively attending to targets have been well studied (see Carrasco, 2011; Driver, 2001; Posner, 1980 for reviews). In recent years, there has been an increased interest in the cognitive mechanisms underlying the suppression of distractor stimuli^1^ (see Chelazzi et al, 2019, Gaspelin & Luck, 2018, van Moorselaar & Slagter, 2020 for reviews). Much of this work has considered how the suppression of distractor stimuli is similar to and diverges from target enhancement. In the present study, we investigated whether spatial cues can facilitate distractor suppression in a similar way to how these cues have been shown to enhance targets.

Recent work has suggested that dedicated cognitive and neural mechanisms are implicated in *attentional suppression* (Chelazzi et al., 2019). That is, it is possible to suppress a stimulus or location to reduce the impact of potentially distracting information on perception. The mechanisms involved in attentional suppression are believed to function proactively (i.e. in anticipation of stimulus onset), reactively (i.e. following attention capture by the distractor) or are triggered by the stimuli (i.e. immediately following the onset of the distractors, prior to attentional capture; see Liesefeld et al., 2024). A number of studies have indicated that proactive suppression can be enhanced through implicit learning about the statistical regularities of the stimuli (see Theeuwes et al., 2022, for a review). For instance, using the additional singleton paradigm (Theeuwes, 1991a) where participants search for a target singleton defined according to a given feature (e.g. a green diamond among green circles) with a distractor singleton defined according to a different feature dimension (e.g. a red circle) appearing on some trials. The difference in performance between distractor singleton present and absent trials is taken as a measure of interference. When the colour of the distractor singleton is predictable across trials, response time costs and saccades towards that singleton are reduced (Vatterott & Vecera, 2012; Won, Kosoyan, & Geng, 2019). Similar effects have been observed using alternative visual search tasks where neutral items are consistently grouped through distinct features from the targets (Stillwell & Vecera, 2019a, 2019b). These findings suggest that (implicit) learning about the features of stimuli can facilitate attentional suppression.

A growing body of research indicates that implicit learning about the likely spatial location of stimuli can also enhance attentional suppression. Where a distractor singleton appeared in one location (65% of singleton present trials; a high-probability condition) more frequently than other locations, response times were reduced compared with a condition in which the location was equiprobable (low-probability condition; Wang & Theewes, 2018a, 2018b; Zhang et al., 2019). Response times remained longer than the distractor singleton absent condition, suggesting that the distractor singleton had not been completely suppressed. The efficiency with which the participant can learn about the distractor singleton location appears to enhance suppression, as the response time benefit tracks with distractor singleton location probability (Lin et al., 2021). In addition to this response time benefit, statistical learning about location reduces eye movements towards that stimulus (Di Caro et al., 2019) and Pd amplitude (distractor positivity; an event-related potential [ERP] measure of reactive suppression; van Moorselaar & Slagter, 2019). It is believed that learning about statistical regularities of distractor location changes the weighting of different locations in the spatial priority map in visual search, meaning that these expected locations will be suppressed. Indeed, the suppression of stimuli in the learned location persists after the probability that the distractor will appear there relative to others is levelled (Britton & Anderson, 2020; Duncan, & Theeuwes, 2020; Sauter et al., 2019). The report of probe letters subsequently presented in locations, which the participant has learnt to suppress, is diminished relative to other locations (Won et al., 2019).

In contrast to the work on implicit learning, evidence regarding the attentional suppression of stimuli following explicit cueing is mixed (see Table 1 for a summary of studies using explicit spatial cues).

**Table 1.** Summary of studies using search tasks investigating attentional suppression following explicit location cues.

Study	Target judgement	Distractor	Measure of interference	Cue-distractor onset interval	Did cues facilitate suppression?
Heuer and Schubö (2020)	Orientation of the line	Singleton distractor	RT, Accuracy, ERP: Pd component	1,600 ms	✓
Leber et al. (2016)	Location of the gap in the circle	Abrupt onset pre-cue	RT, accuracy, identification of masked probe	901–1,201 ms	✓
Munneke et al. (2008)	Identify letter	Target feature matching distractor	RT	1,500 ms	✓
Noonan et al. (2016)	Orientation of the Gabor patch	Randomly oriented Gabors	RT, accuracy, ERPs: P1 N2pc	2,000 ms	×
Van Zoest et al. (2021)	Saccade to target (line oriented 22°)	Line oriented at 67.5°	Saccade RT and deviations. ERP: N2pc, Pd	500–1,250 ms	✓
Wang and Theuwes (2018b)	Orientation of the line	Singleton distractor	RT, accuracy	1,500 ms	×

*Note*. Pd = distractor positivity; ERP = event-related potential; RT = Response Time.

In an early study, participants completed a hybrid flanker-visual search-cueing paradigm (Munneke et al., 2008). Participants were asked to discriminate a target (letter B or F) in an array of letters which included a target feature matching distractor (letter b or f; referred to herein as a foil) which could be congruent (e.g. target B, foil b) or incongruent (e.g. target B, foil f). The upcoming location of the foil was cued in an informative condition, and performance was compared with a non-informative condition in which all locations were cued. The size of the difference of the congruency effect (response times in the incongruent condition minus the congruent condition) was reduced in the informative cue condition compared to the uninformative condition. This suggests that participants were able to proactively suppress the spatial location of the foil in the cued condition. Similar findings have been reported by Leber et al. (2016), who used a spatial cueing paradigm where the target cue also predicted foil location. Response times were faster on valid foil-cued trials compared with invalid cue trials. In a further experiment using a masked probe paradigm, participants were worse when making orientation judgements about a probe stimulus that appeared in the suppressed location. The Pd amplitude has also been found to be reduced following informative spatial cues relative to non-informative ones, supporting attentional suppression (Heuer & Schubö, 2020). However, Heuer and Schubö did not observe any concomitant reduction in response times in singleton distractor present trials when the location was cued. In work combining EEG and eye movement recording, saccades towards the non-target and Pd amplitude were reduced following informative location cues (Van Zoest et al., 2021). Additionally, time-frequency analysis indicated that the magnitude of pre-stimulus alpha power generated by the cue (a measure of proactive suppression) predicted the post-stimulus ERPs. These findings suggest that informative cues can enhance the suppression of stimuli that appear in the cued location.

However, directly contradictory findings have also been observed. Wang and Theuwes (2018b) found no response time benefit following cues to distractor singleton location. In a spatial cueing paradigm, trial-by-trial cue-distractor locations did not decrease response times in distractor-present conditions (Noonan et al., 2016). However, the effect was observed when the distractor location was consistent across a block of trials, supporting a suppression benefit following implicit learning of location. Additionally, there was no modulation of pre-stimulus alpha power by the cues, suggesting against proactive suppression (see Foster & Awh, 2019). Similarly, explicit cues about distractor features (e.g. ‘ignore red’) presented on a trial-by-trial basis do not impact (Stillwell & Vecera, 2019a; Addleman & Störmer, 2022), or can even slow, response times (Moher & Egeth, 2012, Stillwell & Vecera, 2019b) relative to non-informative cues.

In summary, the current evidence regarding explicit cues enhancing attentional suppression is mixed. The goals of the present work are to (a) directly replicate Munneke et al.’s (2008) study using an online experiment and (b), in follow-up experiments, explore the task conditions under which cueing-enhanced distractor suppression is observed (described below). This will provide valuable insight into the nature of attentional suppression following spatial cues. As the beneficial effects of cueing targets on a trial-by-trial basis have been well established (Posner, 1980), work of this nature may also help to unpick critical differences in the function of target enhancement and distractor suppression in selective attention.

### The present study

We sought to provide further evidence on whether foils can be proactively suppressed following spatial cueing and whether there are task conditions that facilitate cued attentional suppression. The primary goal was to replicate Munneke et al.’s (2008) study using online testing to facilitate recruitment of a much larger sample to obtain a more accurate estimate of the effect size of informative versus non-informative cues on the suppression of foils. We also extended the previous study in a number of ways. Firstly, we ran two follow-up experiments with separate samples to investigate cued suppression in greater detail. In Experiment 2, we repeated the experiment with the informative and non-informative cue conditions blocked. This provided a second replication and allowed us to test whether participants require a run of consecutive, informative cued trials to activate a negative template. In Experiment 3, we separated the possible locations of targets and foils (see Di Caroet al., 2019 for a similar approach). This provided a third replication and allowed us to test whether the previously observed effect was due to the cues providing information about the target. In the Munneke et al. (2008) study, the target, foils and neutral letters appeared in four possible spatial locations. Presenting the stimuli in this way effectively reduced the set size in the informatively cued condition, indicating to the participant where the target would not appear. The observed reduction in the response time congruency effect in the informatively cued condition could have been a consequence of the reduction in set size. This incremental approach helped to disambiguate the conditions under which spatially cued suppression is observed.

Second, we used (Bayesian) multilevel modelling to analyse response time data. A major benefit of this approach is that modelling participants as a random effect can reduce the error arising as a consequence of individual differences in participants’ response times emerging from their general processing speed. The Bayesian approach will allow us to measure the relative evidence for the interaction between Congruency and Cue, which was previously observed by Munneke et al. (2008). To improve the efficiency of data collection, we made use of a Bayesian sequential design (Schönbrodt & Wagenmakers, 2018) so that data collection could be halted once a pre-specified threshold of evidence was reached. In addition to hypothesis testing, we provide estimates of likely parameter values given the observed data from the posterior distribution of the Bayesian models.

Third, we supplemented the response time analysis with computational modelling of performance in the informative and non-informative cue conditions. We fitted empirical data to formal models of dynamic spatial selective attention: the dual two-stage processing model (DTSP; Hübner et al., 2010) and the shrinking spotlight model (SSP; White et al., 2011). Both of these models are implemented using a diffusion model framework (Ratcliff et al., 2016) to account for speeded two-choice decision-making. Briefly, the standard diffusion model suggests that decision-making can be understood as a continuous process of noisy evidence accumulation until a criterion (representing a response) is reached. The response time on a task is determined by the time this diffusion process takes to reach a boundary, plus all residual processing latencies, which encompasses all non-decision-making processes (such as the time taken to encode the stimulus and execute the motor response). Diffusion models are widely used in experimental psychology (Evans & Wagenmakers, 2020; Voss et al., 2013), allowing the different processes involved in a response to be decomposed as parameters isolating the efficiency of the stimulus processing (drift rate; μ), conservativeness (boundary separation; β) and non-decision-making processes (*T*_er_). Additionally, diffusion models provide a principled way to combine error and response time data. This is important as these measures are not strongly correlated, suggesting that there are differences in the cognitive processes underlying slowed response times and increased error rates (Hedge et al., 2018).

**Figure 1.**
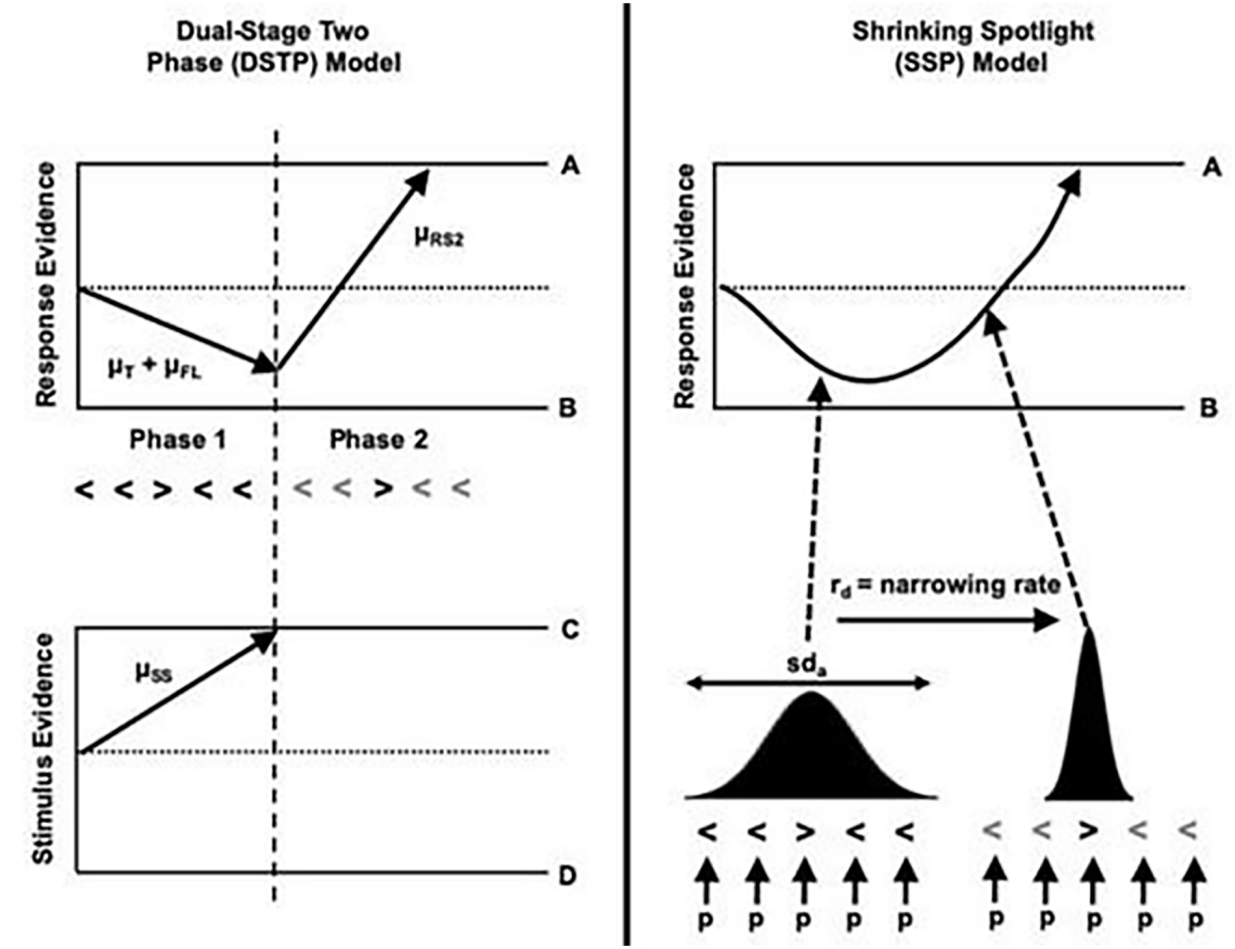
Visual representation of the DTSP model (left) and SSP model (right). For the DTSP model, there is a response and stimulus selection process running in parallel. In the response selection process (top panel), during the first stage, the target and foils both contribute to the overall drift rate. In parallel, there is a stimulus selection process (bottom panel). At the point this hits a boundary (dashed line), the response selection process enters its second stage. At this point, the target is selected, and the diffusion process proceeds rapidly towards the boundary. For the SSP model, the target and foils initially contribute to the overall drift rate. The weighting of the target and flankers is determined by the width of the spotlight of attention, which is characterised as a Gaussian distribution with a mean centred on the target. The distribution gradually narrows on the target, meaning that the foils contribute less to the overall drift rate. *Source*. Figure available at https://flickr.com/photos/150716232@N04/48957578602underCClicense https://creativecommons.org/licenses/by/2.0/. *Note*. DTSP = dual two-stage processing; SSP = shrinking spotlight model.

Both the DTSP and SSP models use the diffusion model framework as a theoretical account of how selective attention unfolds over time. The standard framework is extended as the presence of foils means a response is being made under conditions of conflict. That is, in incongruent conditions, the target and foils provide conflicting information in the response selection process. Each model assumes that the diffusion process is nonlinear. In the early stages, response selection is impacted by the foils as well as the target, whereas in the later stages, more efficient selection of the target takes place. The DTSP and SSP models diverge in whether the selection process occurs in distinct sequential stages or is a continuous process, respectively (see Figure 1 for a schematic of the selection process according to each model).

According to the DTSP model, selection occurs in two distinct phases. In the first phase, attentional selectivity is weaker, meaning that response selection is influenced by both the target and the foils. The drift rate in this early phase (μ_rs1_) is the sum of the drift rate associated with the target (μ_t_) and the foils (μ_fl_). In congruent trials, both μ_t_ and μ_fl_ are positive (i.e. in the direction of the target), whereas in incongruent trials, μ_fl_ is negative. In parallel, there is a late attentional process also characterised as a diffusion process (μ_ss_) aiming to select a single item for further processing. This can reach the upper boundary, meaning the target is selected, or the lower boundary, meaning the foil is selected. When this parallel process finishes before the response selection process (i.e. μ_ss_ beats μ_rs1_ to a boundary), then the response selection enters phase 2. In phase 2, μ_rs2_ begins from where μ_rs1_ finished. Selection is improved in phase 2, and the diffusion process will head rapidly towards the target boundary (although in some instances, if an incongruent flanker has been selected, then the drift rate will be negative). There are seven parameters that can be estimated using this model, which are represented in Table 2. The SSP model also weights the contribution of targets and flankers to an overall drift rate, but here the selection of the target is conceptualised as a continuous process via a gradually narrowing ‘spotlight’ of attention. This spotlight is characterised as a Gaussian distribution with a mean of 0, centred on the target and a standard deviation of *sd*_a_. Initially, attention is diffuse, but the spotlight narrows at a rate of *r*_d_. Five parameters can be estimated using this model, which are summarised in Table 2.

**Table 2.** Summary of the parameters estimated using the DTSP and SSP.

Model	Parameter	Meaning
DTSP	A/B	Response selection boundary height
	C/D	Stimulus selection boundary height
	μ_t_	Target drift rate
	μ_fl_	Foil drift rate
	μ_ss_	Drift rate of stimulus selection
	μ_rs2_	Drift rate of response selection (stage 2)
	*T*_er_	Non-decision time
SSP	A/B	Response selection boundary height
	*p*	Perceptual input from target
	*sd*_a_	Spotlight width
	*r*_d_	Rate of spotlight narrowing
	*T*_er_	Non-decision time

*Note*. DTSP = dual two-stage processing; SSP = shrinking spotlight model.

Both these models account for the common observation that errors in the incongruent condition are more frequent for faster response times (Gratton et al., 1992). This arises as a consequence of the greater weighting of the foil in the drift rate early in the response selection process. It is a matter of ongoing debate as to whether selectivity is a continuous or discrete process, with model comparison studies variously showing the DTSP (Hübner & Töbel, 2012; Servant et al., 2014) or SSP (White et al., 2011; White et al., 2018) to provide a superior fit to empirical data. Additionally, studies using methods that continuously track the participants’ movements when making a response have provided support for continuous (using mouse tracking; Kinder et al., 2022) and discrete (using electromyography; Servant et al., 2015) selection.

To date, no studies of active suppression have made use of these attentional models. However, as described above, this allows the decision-making process involved in the participants’ response to be decomposed into parameters with psychologically meaningful interpretations. This can help to unpick any differences between informative and non-informative cued conditions, particularly whether speeded responses in the informatively cued condition reflect attentional suppression, attentional enhancement or a less cautious response style.

Finally, we incorporated web-based gaze tracking (Papoutsaki et al., 2016) during the experiment. Participants were instructed to centrally fixate during the task. The participants had the opportunity to direct their gaze away from the foil location during the duration from the onset of the cue to the search array (1,500 ms). If gaze is averted in this way, then the visual representation of the foil would be degraded in informative trials compared to non-informative trials, and the response time difference could reflect retinal eccentricity rather than attentional suppression. We explored whether there were any systematic differences in how participants maintained central fixation between the informative and non-informative cueing conditions.

#### Hypothesis

In all three experiments, we anticipated that the difference in response times between incongruent and congruent trials would be smaller in the informative cue condition compared to the non-informative cue condition (i.e. an attenuated or partially attenuated interaction between congruency and cue). We fitted the DTSP and SSP models to the empirical data, and the parameter estimates were used to interpret the psychological processes involved in any differences between the conditions.

## Methods

### Participants

Participants were recruited via Prolific (https://www.prolific.co/) and were required to complete the task using their laptop or desktop computer. Inclusion criteria were as follows: based in the UK or Ireland, aged 18 to 40 years, access to a functioning webcam, no forms of neurodivergence, neurological conditions or visual impairments and an approval rating of 95% on Prolific, having completed more than 50 studies previously. In Experiment 2, an additional inclusion criterion was that participants had not taken part in Experiment 1, and in Experiment 3, the participants had not taken part in Experiments 1 or 2.

The study was ethically approved (University of Sheffield, Reference Number 050440). All participants were asked to complete an online consent form before being able to access the study.

#### Sample size

##### Recruitment

This study used a sequential Bayes factor design with a maximum sample size (Schönbrodt & Wagenmakers, 2018). The decision rule for stopping was based on the relative evidence comparing the interaction model (Congruency * Cue) with the model with the main effects (Congruency + Cue). The a priori minimum sample size (*n*_min_) was 80 participants, and the maximum (*n*_max_) was 200. Interim analysis was conducted every ~20 participants (between *n*_min_ and *n*_max_). The threshold to stop testing at each interim analysis was whether the *BF*_10_ >10 or <1/10, which means that the data were 10 times more likely under the model including the interaction relative to the main effects (or vice-versa if *BF*_10_ < 1/10).

### Exclusions

There were three criteria to identify participants who were not paying attention or were unable to perform the experiment appropriately and whose data were removed prior to analysis.

1. Failing 50% of the catch trials (described below)
2. Accuracy <85% on the task overall
3. Response time trimming resulted in the removal of >40% of trials. Response times were trimmed on trials where response times <200 ms RT >1,200 ms to exclude anticipation and miss trials.

In Experiment 1, 199 participants completed the study. The final sample size following exclusions was 189 (mean age = 33.02, *SD* = 5.26; gender: 103 male/man, 86 female/woman; sex: 86 male/man, 103 female/woman; ethnicity: 137 White, 26 Black, 16 Asian, 6 Multiracial, 4 Other).

In Experiment 2, 193 participants completed the study. The final sample size following exclusions was 183 (mean age = 30.71, *SD* = 5.29; gender: 83 male/man, 100 female/woman, sex: 83 male, 100 female, ethnicity: 147 White, 14 Black, 13 Asian, 8 multiracial, 1 other).

In Experiment 3, 194 participants completed the study. The final sample size following exclusions was 182 (mean age = 31.13, *SD* = 5.51; gender: 91 male/man, 90 female/woman, 1 transmale/transman, sex: 91 male, 91 female, ethnicity: 113 White, 28 Black, 29 Asian, 9 multiracial, 3 other). See deviations from pre-reg below for explanation of the discrepancy between pre-registered and final sample sizes.

The breakdown of the number of participants who were excluded by each of the criteria above is listed in the results.

### Stimuli and materials

Experiment 1 is a replication of Munneke et al.’s (2008; Experiment 2) study. All experiments are available on Gorilla’s Open Materials page: https://app.gorilla.sc/openmaterials/958738.

**Figure 2.**
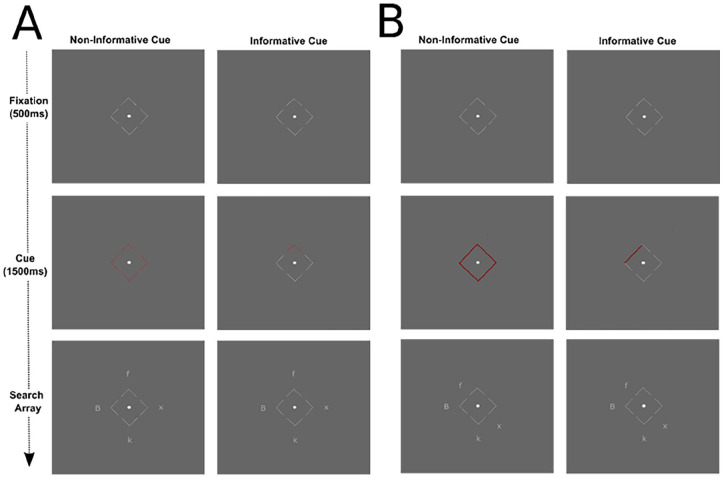
A schematic of an incongruent trial in the informative cue and non-informative cue conditions in Experiments 1 and 2 (A) and Experiment 3 (B).

Participants completed a hybrid flanker-visual search-spatial cueing paradigm with a set size of four. On each trial, participants identified a target capital B or F by pressing the corresponding key on their keyboard. A foil was presented on each trial, which was either congruent (e.g. target B and foil b) or incongruent (e.g. target B and foil f) with the target. The other two items in the array were x and ks. These were randomly selected for each trial in advance, fixed between participants and identical for the informative and non-informative conditions. Participants were asked to centrally fixate a target dot surrounded by four arrows pointing to the possible locations of the stimuli. See Figure 2 for schematics of the search array.

During the task, eye movement recordings were measured using WebGazer (Papoutsaki et al., 2016) controlled using the Eye Tracker 2 app in Gorilla. WebGazer estimates gaze location by detecting the pupils through the participants’ webcam. Gaze location estimates were sampled at a rate of 60 Hz.

### Procedure

After completing the consent and demographic information, participants were presented with a video explaining the task. Before beginning the experiment, they were asked to complete the virtual chin rest task (Li et al., 2020) to obtain an estimate of the distance from the screen.

The participant then began the experiment. A schematic of the sequence on a trial in each Experiment is presented in Figure 2.

In Experiment 3, the possible target and foil locations were distinguished based on spatial location (see also Di Caro et al., 2019). There were four possible target locations (identical to Experiments 1 and 2) and four possible foil locations (see Figure 2B). The foil locations were next to the four sides of the fixation square created by the contours of the arrows. The set size remained as four, with neutral stimuli randomly appearing in either possible target or foil locations.

In each experiment, the participants were presented with the fixation screen for 500 ms followed by the cue for 1,500 ms. In Experiments 1 and 2, in the informative cue condition (50% of trials), the arrow pointing to where the foil appeared turned red, while the other sides remained grey. In the non-informative cue condition (50% of trials), all the arrows turned red. In Experiment 3, the informative cue was a red line along the length of the fixation square in the informative cue condition (50% of trials). In the uninformative cue condition (50% of trials), the entire square turned red (see Figure 2B). Immediately following the cue, the search array was presented until a response was recorded. Gaze location estimates were recorded during the presentation of the search array. After responding, participants pressed space when ready to continue. Participants were instructed to respond ‘quickly but accurately’ and to maintain central fixation throughout the task. They were not given any specific information about the cue.

Participants completed 16 blocks of 19 trials, giving 304 trials in total. This included 16 catch trials in which all the items in the array were o, the participant was instructed to withhold a response when the target was absent. The catch trials were included so that any low-motivation participants responding as quickly as possible could be rejected (Exclusion criteria 1). Before beginning the task, participants were asked to complete a five-point validation procedure, which was repeated between each block. For every four blocks, there was an enforced break of 9,000 ms before the participant could continue.

In Experiments 1 and 3, informative and non-informative cue trials were fully interleaved, as were target (B or F) congruency (congruent or incongruent) and the location of the target, foil and neutral letters. In Experiment 2, the cue condition was blocked. The experiment was split into four epochs of 76 trials, two epochs in which the participant was presented with the informative cue only, and two epochs in which the participant was presented with the non-informative cue only. All other elements (target identity, congruency, target and foil locations) were randomised as in Experiments 1 and 3. The epochs were presented in a random order for each participant. The break and eye tracking recalibration occurred every 19 trials, as with Experiments 1 and 3.

At the end of each experiment, participants completed a test of cue-foil location association. The test consists of an image of the fixation square with an x either in the bottom left or top right location. The participant was asked, ‘When you saw this cue, how often was the foil where the x is?’ They slide a scale from Never to Always with a value from 0 to 100 appearing above the scale. There were four questions: two for the informative cue (one valid and one invalid) and two for the non-informative. There were four possible orders to the questions, which were counterbalanced across participants.

On summarising the differences between experiments, Experiment 1 was a direct replication of Munneke et al. (2008). Experiment 2 repeated Experiment 1, except that the cue conditions were blocked rather than interleaved. Experiment 3 separated the possible locations of the target and foils, and cue conditions were interleaved.

### Analysis

All data preparation and analysis were conducted in R (R Core Team, 2023). Raw data, analysis scripts and Supplemental Material are available on the study Open Science Framework page (https://osf.io/zqfm3/).

### Response times

Response times were trimmed prior to analysis to remove responses <200 and >1,200 ms to omit anticipation and miss trials. We fitted Bayesian General Linear Mixed Models using brms (Bürkner, 2017) to the response times on accurate trials. Brms is a front-end for Stan which fits using Hamilton MCMC methods. Participant-averaged response times were the outcome variable modelled as a Gaussian distribution with Congruency (Congruent, Incongruent) and Cue (Informative vs. Non-informative) as the predictors, plus participant as a random effect (RT ~ Congruency * Cue + (1|Participant)). We used default regularising priors in brms and 4 chains drawing on 5,000 samples, with the first 2,000 samples used as warm-ups. The chains were visually inspected for convergence (see Supplemental Material S1) and $\overset{⌢}{R}$ was close to 1 (Experiment 1 = 1.01, Experiment 2 = 1 and Experiment 3 = 1). Firstly, the critical test of our hypothesis is whether differences in response times between the congruent and incongruent conditions are modulated by the cue. To test this hypothesis, we calculated the Bayes factor (using the bayestestR package, Makowski et al., 2019) as a measure of the relative evidence for the model including the interaction (RT ~ Congruency * Cue + (1|Participant) compared with main effects model (RT ~ Congruency + Cue + (1|Participant). As described in the recruitment section, Bayes Factor values of either *BF*_10_ = 10 or *BF*_10_ = 1/10 are the evidence thresholds to stop testing at the interim analysis. Second, the beta estimate for the interaction term and its 95% credible interval was calculated from the posterior samples. We used a highest density interval (HDI) plus region of practical equivalence (ROPE) approach (HDI + ROPE, Kruschke, 2018) to assess whether estimated parameter values fell within a range that might be considered equivalent. The ROPE was defined as −0.1 to 0.1, and we reported whether >89% of the HDI fell inside or outside of the ROPE.

### Qualitative visual assessments

To assess active suppression, it is important to confirm that the to-be-suppressed stimuli can impact target performance using that paradigm (Wöstmann et al., 2022). As a positive control, we plotted individual participant congruency effects across conditions. The congruency effect is calculated as *median* RT_Incongruent_ – *median* RT_Congruent_; we should expect to see a positive congruency effect in most participants (i.e. *CE* > 0 in 75% of participants). Data from pilot studies met these criteria (https://osf.io/nb9sv).

We calculated conditional accuracy functions (CAFs, Gratton et al., 1992) using the flankr R package (Grange, 2016). CAFs show the accuracy in congruent and incongruent trials when response times are sorted into five bins. The typical observation is that errors are more frequent in the incongruent condition for the fastest response times, and at slower response times, the accuracy on congruent and incongruent trials converges. If cues enable proactive suppression of foils, then the CAF should be flatter in the informative condition.

#### Model fitting

The DSTP and SSP models were fit to individual participant data using the flankr R package (Grange, 2016).^2^ The models were fitted to (trimmed) RT and accuracy distributions using the Nelder-Mead algorithm to find the best set of parameters which minimise the likelihood ratio chi-squared statistic to the empirical data (see Grange, 2016, for full details). For each model, one set of parameters simultaneously describes performance on both congruent and incongruent trials. The models were fit separately to both levels of the cue condition (informative vs. non-informative); all parameters in Table 2 were free to vary across both levels of the cue.

To avoid local minima during parameter estimation, we used the recommendations of Grange (2016) whereby parameter optimisation occurred across two steps. In the first step, a broad search of the parameter space was conducted by starting the fit routine from 50 random starting points. The starting points were sampled from a normal distribution with a mean of the flanker-default starting values^3^ and a standard deviation of ⅕ of these default values. During this first step, 1,000 trials were simulated on each iteration of the fit routine. In a second step, we selected the best set of parameters from the first step and used them as starting parameters for a second search routine, simulating 50,000 trials per iteration of the fit routine. The final best-fitting parameters from this second step were stored as the final parameters. Goodness of fit was assessed via visual assessments of the fit between empirical and predicted data for the best-fitting model (see Supplemental Material S2). Specifically, we inspected the model fits via construction of QQ-plots, which plot simulated response time and accuracy from the best-fitting parameter values against observed data for the overall accuracy and for the 25th, 50th and 75th percentiles of the response time distribution. We also used the binned Bayesian information criterion to determine which model provides the best fit to the observed data.

The best-fitting parameters of each model were used to interpret the cognitive processes underlying any differences between the conditions, with the interpretations associated with each parameter provided in Table 1. Of particular interest was whether measures of interference from the foils (see White et al., 2018) are reduced in the informative cue condition. For the SSP model, the spotlight width (*sd*_a_) and shrinking rate (*r*_d_) can be used to calculate a measure of interference time $\left(\frac{sd_{a}}{r_{d}}\right)$ , which can be interpreted as the time taken to suppress the foils. For the DTSP model, a similar measure can be calculated using the drift rate associated with the flankers (μ_fl_) and the drift rate for the stimulus selection process $\left(\mu_{\mathrm{SS}};\;\frac{\mu_{\mathrm{fl}}}{\mu_{\mathrm{ss}}}\right)$ . If the informative cue enables foils to be suppressed, then interference time should be reduced. Alternatively, if the cues encourage a less conservative response strategy, then boundary separation (A/B) should be reduced in the informative condition relative to the non-informative. If the processing of the target, then measures of target processing should be increased in the cue conditions (*p* for the SSP model or μ_t_ for the DTSP). A series of Bayesian regressions were performed with model parameter as the outcome variable and cue condition as the predictor (Parameter ~ Cue + (1|Participant)).

##### Cue association learning

To assess participants’ awareness of the association between cue and foil location, we plotted density functions of ratings on the informative and non-informative cue conditions. If participants learnt the association between foil and cue in the informative condition, then the distribution should be shifted towards 100 when the cue is valid and 0 when invalid. In the non-informative cues, both should overlap around 25.

##### Maintenance of central fixation

To assess the extent to which participants maintained central fixation to a similar degree between conditions, we plotted gaze location estimates in the informative and non-informative cue conditions. Gorilla normalises the *x* and *y* coordinates of gaze location to the participant’s screen size. Firstly, we plotted heat maps of these screen-normalised gaze location estimates across the cue conditions (see Supplemental Material S3). Second, we analysed the percentage occupancy in an area of interest. We defined the area of interest as the area around the fixation point and arrows, screen-normalised coordinates *x* (0.35:0.65) and *y* (0.35:0.65). For each participant, we calculated the proportion of trials in which ⩾50% of the participants’ gaze location estimates fell inside this area of interest (AOI) during the first 500 ms of the trial. These were then compared between the informative and non-informative cue conditions using raincloud plots.

##### Deviations from pre-registration

We made the following deviations from the pre-registration. We calculated the Bayes factor using the BayesFactor package rather than BayesTestR as pre-registered. This was due to the BayesTestR incompatibility with uninformative priors.

There are minor discrepancies from the pre-registration in how the sequential analysis was conducted. We pre-registered interim analyses at fixed intervals (*n* = 20) once we reached *n*_min_, However, in practice, interim analyses occurred approximately every 20 participants. This was due to slight over- or under-recruitment caused by withdrawals and incomplete submissions on Prolific. Additionally, the final sample sizes in each experiment fell slightly short of *n*_max_ as recruitment slowed considerably. We speculate that we may have approached the saturation point for active, eligible participants on Prolific, especially given our requirement that participants use a webcam during the study. We do not believe that stopping early has meaningfully affected the study outcomes. To support this, we conducted post hoc simulations estimating the Bayes Factor if we had reached *n*_max_. These simulations are reported in Supplemental Material S1 and suggest early stopping had no effect on the direction of the findings and minimal impact on evidential strength.

We have added a non-pre-registered exploratory analysis in which we conduct a content analysis of participants’ open-ended responses to the question ‘Any Other Comments About What the Cue Might Have Been For?’ Participants were presented with this question at the end of the assessment of Cue Learning. We decided to include this analysis as the quantitative data appeared to show that overall, participants had not learnt the association between the cue and foil location.

## Results

Exclusion of participants:

Experiment 1
1. Zero participants were removed for failing catch trials.
2. Three participants were removed for low accuracy.
3. Seven participants were removed for too many trials being trimmed.
Experiment 2
1. Zero participants were removed for failing catch trials.
2. Nine participants were removed for low accuracy.
3. One participant was removed for too many trials being trimmed.
Experiment 3
1. Zero participants were removed for failing catch trials.
2. Eight participants were removed for low accuracy.
3. Four participants were removed for too many trials being trimmed.

### Response time data

**Figure 3.**
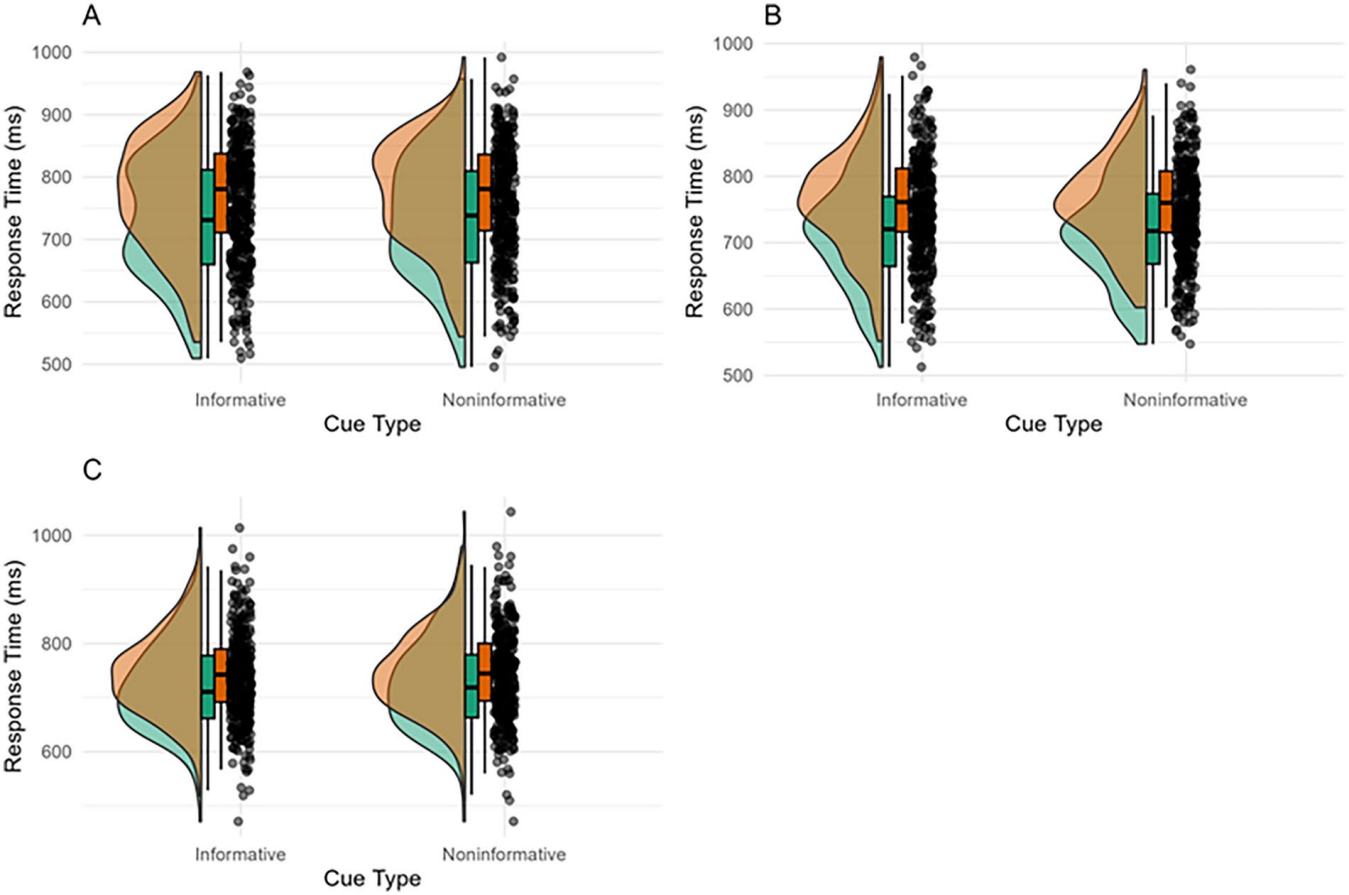
A Raincloud plot illustrating the response time (ms) on congruent (green) and incongruent (orange) trials in the informative and non-informative conditions for Experiment 1 (A), Experiment 2 (B) and Experiment 3 (C). See Supplemental Material S1 for an interaction plot displaying sample average response times in each condition.

Participant-averaged response times in each experiment are displayed in Figure 3. As can be seen in Figure 3, there was a congruency effect in both the informative and non-informatively cued conditions. The positive control was passed in each experiment, with >75% of participants showing a positive congruency effect. In Experiments 1 and 3, responses were slower in the informative cue condition. Importantly, the congruency effect was not modulated by cue.

**Figure 4.**
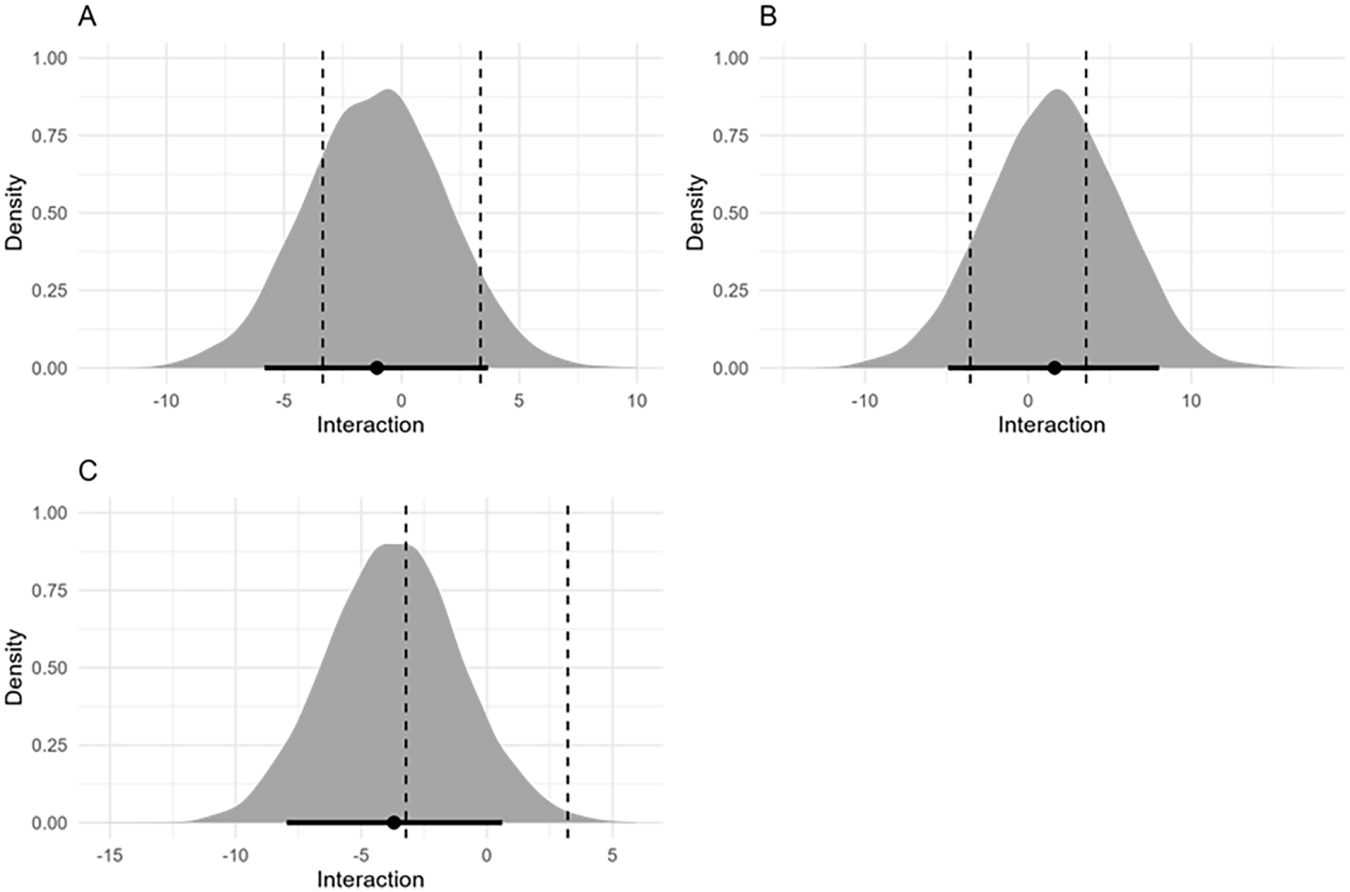
Interaction term beta estimate with 89% highest density interval for Experiment 1 (A), Experiment 2 (B) and Experiment 3 (C). The dashed lines represent the boundaries of the region of practical equivalence.

For the RT analysis, there was a large effect of Congruency (Experiment 1: β_incongruent_ = 38.58 [95% credible interval 34.55, 42.74], Experiment 2: β_incongruent_ = 40.07 [34.56, 45.89], Experiment 3: β_incongruent_ = 23.98 [20.33, 27.67]), negligible effects of Cue Type (Experiment 1: β_non-informative_ = 5.44 [1.29, 9.65], Experiment 2: β_non-informative_ = −1.63 [−7.32, 3.99], Experiment 3: β_non-informative_ = 1.89 [1.83, 9.22]) and of the interaction between Cue Type and Congruency (Experiment 1: β_incongruent:non-informative_ = −1.08 [−6.95, 4.71], Experiment 2: β_incongruent:non-informative_ = 1.60 [−6.46, 9.48], Experiment 3: β_incongruent:non-informative_ = −2.65 [−8.86, 1.57]). For each experiment, the HDI + ROPE analysis of the interaction between Cue Type and Congruency was inconclusive according to our pre-registered criteria (>89% of the HDI did not fall inside or outside of the ROPE, see Figure 4). For Experiment 1, the posterior distribution for the interaction gave a 89% HDI of −5.83 to 3.62. The ROPE was defined as −3.35 to 3.35. We found that 71% of the 89% HDI fell inside the ROPE. For Experiment 2, the 89% HDI was −4.91 to 8.03, the ROPE was defined as −3.55 to 3.55. We found that 58% of the 89% HDI fell inside the ROPE. For Experiment 3, the 89% HDI was −7.96 to 0.61, and the ROPE was defined as −3.22 to 3.22. We found that of the 89% HDI, 42% fell inside the ROPE.

Finally, for each experiment, the Bayes Factor indicated stronger support for the main effects model compared to the model including the interaction term. Experiment 1: The Bayes Factor comparing the interaction term and main effects model was *BF* = 0.11, meaning that the main effects model was 9.09 times more likely than the interaction model given the observed data. Experiment 2: The Bayes factor comparing the interaction term and main effects model was *BF* = 0.13, meaning the main effects model was 7.44 times more likely. Experiment 3: The Bayes factor comparing the interaction term and main effects model was *BF* = 0.30, meaning the main effects model was 3.33 times more likely.

### Conditional accuracy function

**Figure 5.**
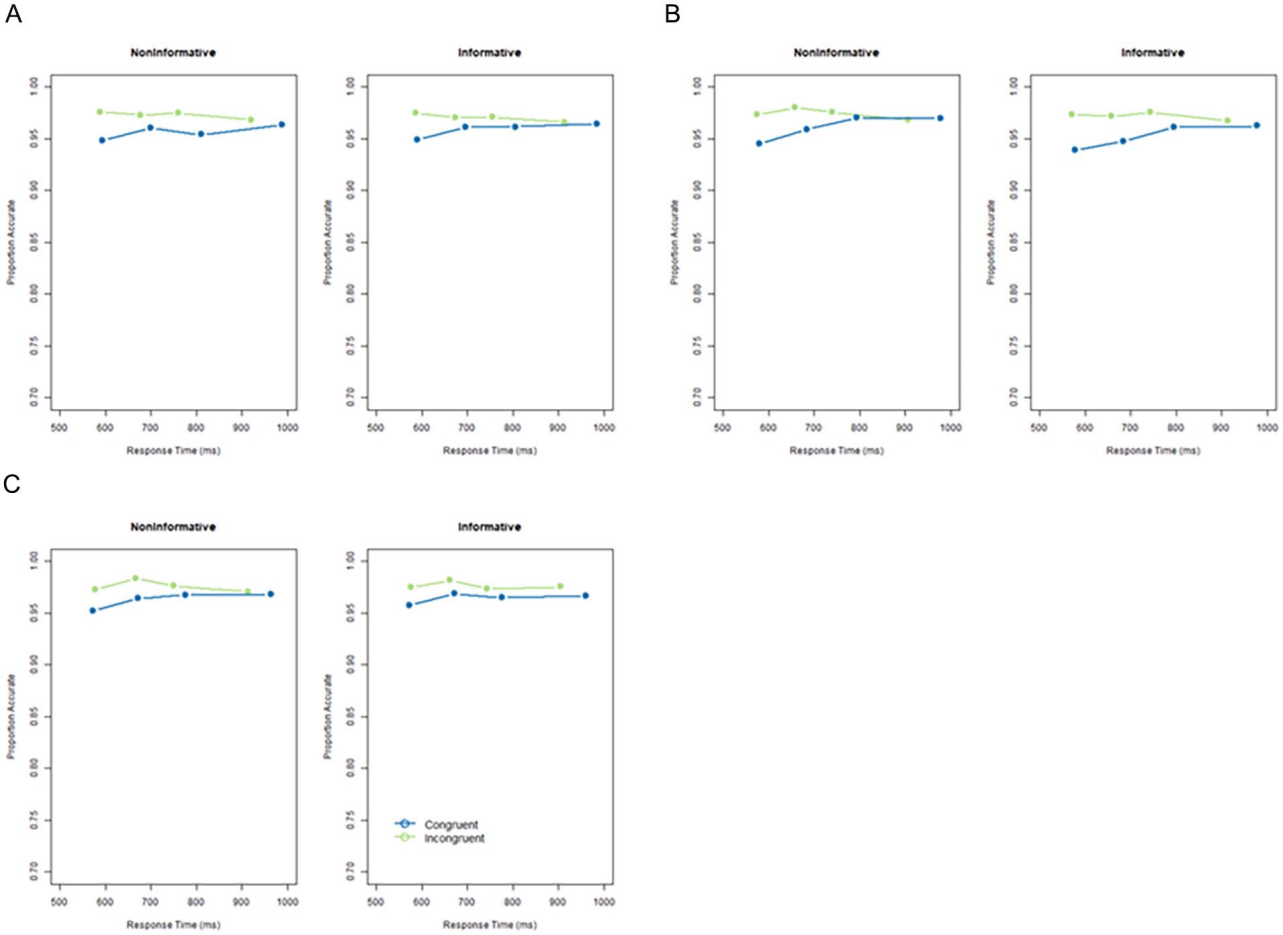
Conditional accuracy functions in the non-informative and informative conditions in Experiment 1 (A), Experiment 2 (B) and Experiment 3 (C). Congruent trials are given in blue and incongruent trials in green. Conditional accuracy functions display the proportion of accuracy in each quartile of the response times in each condition.

CAFs for each cue condition in each Experiment are displayed in Figure 4. In each experiment, the typical effect was observed in both the cued and uncued conditions, whereby the difference in accuracy between the congruent and incongruent conditions was largest for the fastest RT (Figure 5).

### Model parameter estimates

**Figure 6.**
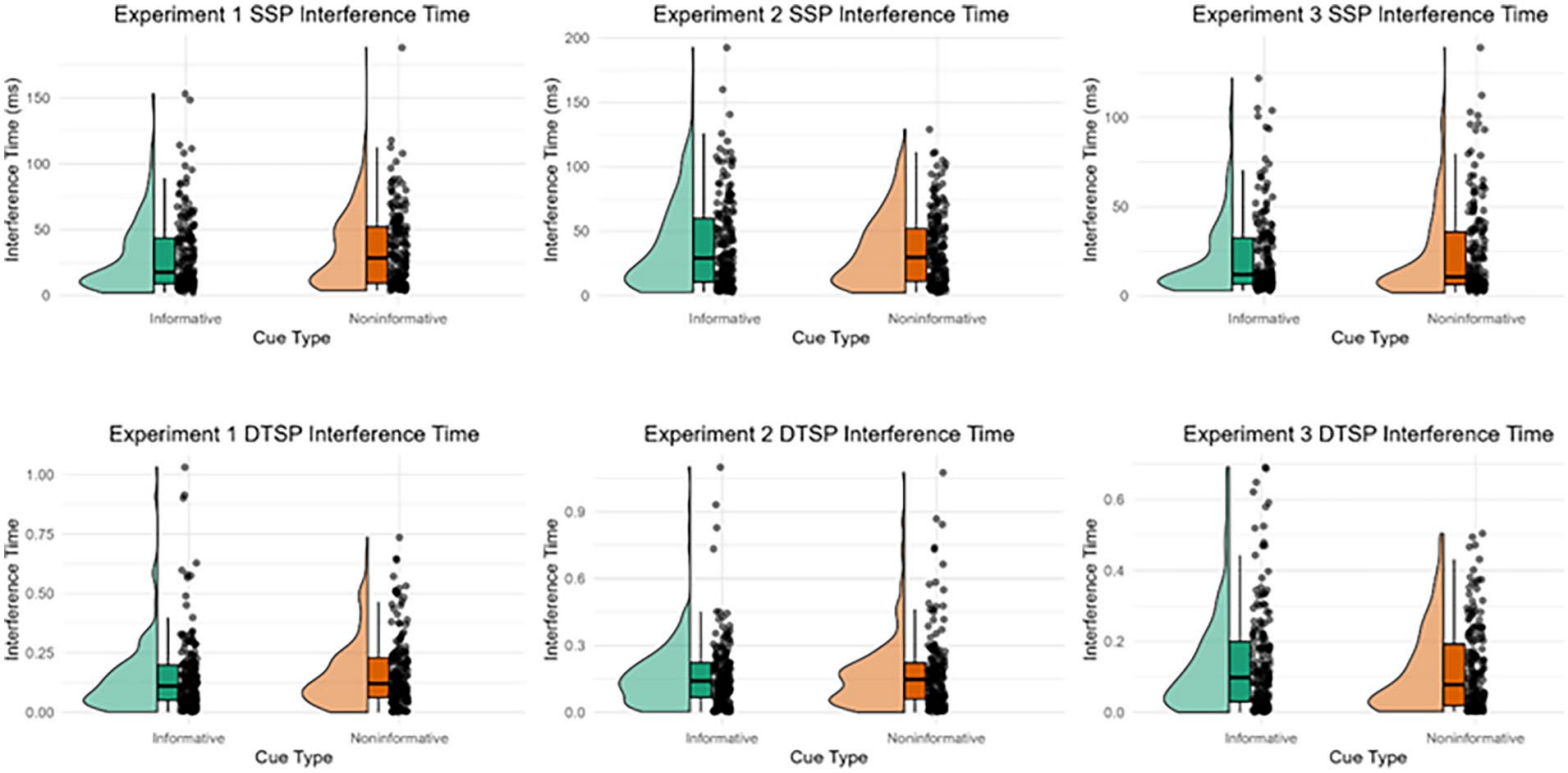
Raincloud plots displaying the interference time estimated using the shrinking spotlight model (SSP; row 1) and dual two-stage processing model (DTSP; row 2) in the informative and non-informative conditions for each experiment.

The estimates of Interference Time using the SSP and the DTSP model in the informative and non-informatively cued conditions of each experiment are displayed in Figure 6. The Interference Time was not modulated by the cue in any of the experiments.

This was confirmed using regression models (see Table 3). Please see Supplemental Material S2 for the plots of the other parameter estimates from each model.

**Table 3.** Results of Bayesian regression with cue condition as the predictor and each of the estimated parameters from the SSP and DTSP in each experiment.

Model	Parameter estimate	β_non-informative_ [95% BCI]
Experiment 1		
SSP	Interference time	4.83 [−0.42, 10.06]
	*p*	<−0.01 [−0.01, 0.01]
	A	<−0.01 [−0.01, 0.01]
	*T*_er_	<0.01 [−0.01, 0.02]
DTSP model	Interference time	0.02 [−0.01, 0.04]
	µ_t_	0.01 [−0.01, 0.02]
	μ_rs2_	−0.01 [−0.07, 0.05]
	A	−0.01 [−0.01, 0.01]
	C	<0.01 [<−0.01, 0.01]
	*T*_er_	−0.01 [−0.02, 0.01]
Experiment 2		
SSP	Interference time	−3.87 [−9.45, 1.73]
	*p*	< 0.01 [−0.01, 0.02]
	A	< 0.01 [−0.01, 0.01]
	*T*_er_	0.01 [−0.01, 0.02]
DTSP	Interference time	0.01 [−0.02, 0.04]
	µ_t_	<−0.01 [−0.01, 0.01]
	μ_rs2_	0.04 [−0.01, 0.10]
	A	< 0.01 [<−0.01, 0.01]
	C	<−0.01 [0.01, 0.01]
	*T*_er_	<−0.01 [−0.02, 0.01]
Experiment 3		
SSP	Interference time	1.33 [−3.22, 5.85]
	*p*	−0.01 [−0.02, <0.01]
	A	<−0.01 [−0.01, 0.01]
	*T*_er_	<0.01 [−0.01, 0.01]
DTSP	Interference time	−0.01 [−0.03, 0.01]
	µ_t_	<−0.01 [−0.01, 0.01]
	μ_rs2_	−0.03 [−0.07, 0.01]
	A	<−0.01 [−0.01, 0.01]
	C	<−0.01 [−0.01, <0.01]
	*T*_er_	<0.01 [−0.01, 0.01]

*Note*. BCI = Bayesian credible interval; DTSP = dual two-stage processing; SSP = shrinking spotlight models.

### Cue association learning

**Figure 7.**
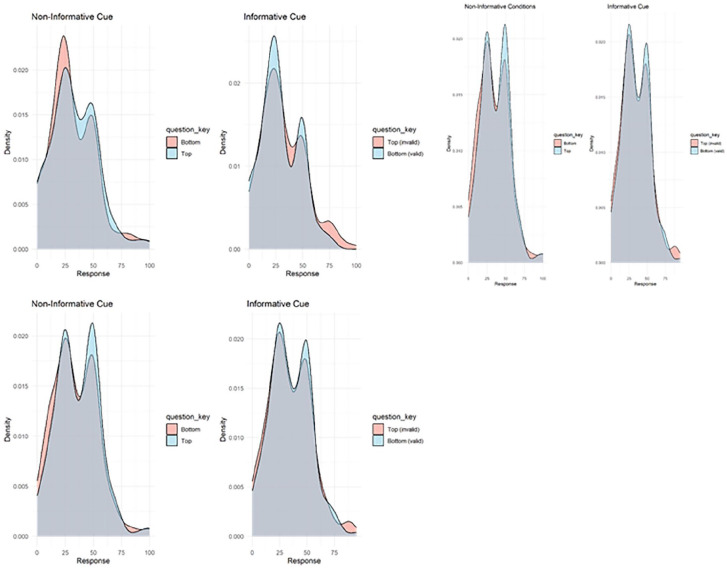
Participants were asked, ‘When you saw this cue, how often was the foil where the x is?’ with an x at the top (pink) or bottom (blue) of a photo of an empty search array. In the informative cue questions, the cue was pointing to the bottom. The correct responses in the informative cue condition would be top = 0, bottom = 100, and in the non-informative condition, top = 25, bottom = 25.

The density plots displaying the participants’ cue association learning are displayed in Figure 7. The distributions are similar in the informative and non-informative conditions, suggesting that the participants did not learn the association between cue and foil location.

### Exploratory analysis

As an additional, non-pre-registered, exploratory analysis, we conducted a content analysis (Mayring, 2014) on participant responses to the open-ended question ‘Any other comments about what the cue might have been for?’ Author D.P. developed the codebook inductively based on responses given in Experiment 1, then D.P. and E.M. independently coded the entire dataset. A total of 236 responses received a code (43% of the sample). Inter-rater reliability was good (*k* = 0.78, *p* < .001). Reported codes (see Table 4) are based on the final coding agreed in a consensus meeting. The most frequent codes were *the cues themselves were to distract you* (35.62% of responses) and *did not notice any associations* (32%). Only 4.72% of responses received the correct code: *the cues told you where the foil would appear*.

**Table 4.** Counts for each code in response to the question ‘Any other comments about what the cue might have been for?’.

Code	Count (%)
The cues told you where the foil (lower case f/b) would appear (correct answer)	11 (4.72)
The cues themselves were to distract you	83 (35.62)
Did not notice any associations	75 (32)
Cues were random	10 (4.29)
The cues told you where the target (upper case F/B) would appear	7 (3)
Other	47 (20.17)

### Maintenance of central fixation

**Figure 8.**
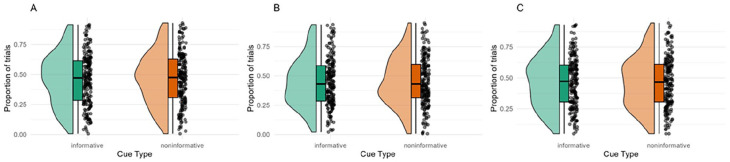
Raincloud plot displaying the proportion of trials in which ⩾50% of the participants’ gaze location estimates fell inside the AOI during the first 500 ms of the trial. *Note*. AOI = area of interest.

To compare eye gaze locations between conditions, the proportion of trials in which ⩾50% of the gaze location estimates fell inside the AOI during the first 500 ms of the trial. As can be seen in Figure 8, there were no systematic differences in eye gaze location between the conditions in any of the experiments.

## Discussion

In the present study, we used a hybrid visual search-flanker-cueing paradigm (cf Munneke et al., 2008) to investigate whether spatial cues facilitate distractor suppression. Across three experiments with a total of 554 participants, we did not observe credible evidence that spatial cues impacted attentional suppression.

Across all three experiments, we observed a typical response time congruency effect for variants of the flanker paradigm (Eriksen & Eriksen, 1974). CAFs showed a similar pattern to what is usually observed on a flanker task, whereby the congruency effect was largest for shorter response times and gradually reduced (Gratton et al., 1992; Hübner & Töbel, 2019). This indicates that the foils were capturing attention and interfering with responses to the target. However, this congruency effect was not modulated by cue, either when informative and non-informative cues were interleaved (Experiment 1 and 3) or blocked (Experiment 2). In Experiment 1 and Experiment 3, there was a small effect of Cue which was indicative of participants responding more slowly overall in the informative cue condition. In Experiment 1 and Experiment 3, cue type was interleaved, meaning that within a block the cue which was presented on each trial was unpredictable. A previous study using a similar hybrid-visual search task (cf Munneke et al., 2008) but using feature rather than spatial cues (‘ignore red’), observed increased response times and error rates following informative cues (Moher & Egeth, 2012). This may indicate that, when cue type is unpredictable on a trial-by-trial basis, informative, central cues can capture attention and slow visual search.

Additionally, we fitted models of performance on conflict tasks, the SSP model (White et al., 2011) and the DTSP model (Hübner et al., 2010), to response time and error data. We used these models to extract the interference time, which is a measure of the time taken for participants to suppress the foils/focus attention towards the target. There was no credible evidence that the cue type modulated interference time across the Experiments. This provides further evidence against cueing enhanced attentional suppression in the present study. Furthermore, we did not observe any credible differences in boundary separation, non-decision time or drift rate between conditions. This suggests that between the informative and non-informative cue conditions, participants did not adjust their response criterion, process the stimuli/prepare the motor response differently, nor were targets processed more effectively in the presence of informative cues.

The experiments conducted in the present work provide a valuable insight into the nature of distractor suppression. Experiment 1 was a direct replication of Experiment 2 from Munneke et al. (2008), where cues facilitated distractor suppression. We did not replicate this finding; in fact, the evidence against an effect of cueing on distraction suppression was strongest in this study (through the relatively highest Bayes Factor and the greatest proportion of the HDI falling in the ROPE). There are some key differences from the original method, which could account for the disparity in findings. Firstly, the current study was conducted online rather than in laboratory. We took measures to mitigate against variability in viewing conditions by including a virtual chinrest at the beginning of the experiment and recording participants’ eye movements during the task. However, it may be that the current findings are a false negative, as the reduced control over trial-by-trial viewing conditions may have increased the variability in the effect of the cues. On the other hand, online testing allowed for the recruitment of a considerably larger number of participants (*n* = 14, in Munneke et al., 2008), and it may be that the previously observed effect was a false positive arising through sampling error. Second, the current study included eye movement recording through webcam gaze tracking. We included eye movement recording so that we could discount the possibility that the previously observed effect was a consequence of participants moving their eyes away from the location of the foil rather than an attentional suppression effect. The eye movement data are noisy and does not suggest differences between conditions. However, the presence of eye movement recording, including recalibrations after each block, may have placed greater weight on the instruction to centrally fixate than in Munneke et al.’s (2008) study. We tentatively suggest that eye movements away from the upcoming distractor location following the informative cue might explain the difference in findings and might account for some of the variation between studies of spatial cueing. A final consideration is whether the participants were made aware of the cue-distractor association in advance.^4^ In Experiment 1 of the Munneke et al. (2008) study, participants were ‘instructed to actively use this information’ about the cue (p. 102). Although this was not specifically reported for Experiment 2, the version replicated in the present study, it is reasonable to assume similar instructions were provided. Re-reviewing the related work on explicit spatial cues (described in Table 1), we found two additional instances where explicit instructions about the cue were reported in the manuscript. In one study, cueing-enhanced distractor suppression was observed: ‘Participants were fully instructed about the meaning and validity of these cues and were encouraged to make use of the information provided by the predictive cues’ (Heuer & Schubö, 2020, p. 2114). In the other, no cueing effect was observed: ‘Participants were provided with foreknowledge of the location of the target (Target Cue), distractor (Distractor Cue), or no predictive information (Neutral Cue) via a central cue. This cue condition was instructed to subjects at the beginning of each block’ (Noonan et al, 2016, p. 1798). Participant awareness of the cue-distractor association is discussed further below.

In the present study, we have not observed any credible differences in response times or diffusion model parameters reflective of cued attentional suppression. Note that according to our pre-registered criteria for HDI plus ROPE, the evidence regarding the interaction might be considered inconclusive. Nonetheless, this work has provided a valuable contribution to the literature on attentional suppression, suggesting that explicit, spatial cues do not facilitate suppression. This is in line with previous work, which has not supported the trial-by-trial suppression of distractors following spatial (Noonan et al., 2016; Wang & Theuwes, 2018b) or feature cues (Addleman & Störmer 2022; Moher & Egeth, 2012; Stillwell & Vecera, 2019a, 2019b). The previous suggestion that cueing-enhanced attentional suppression only occurs when target-distractor similarity is high (Van Zoest et al., 2021) was not supported here, where the target and foil shared both identity and featural properties. These findings indicate that, unlike the way cues enhance the selection of target items (e.g. Posner, 1980; Theeuwes, 1991b; Wolfe et al., 1989), they do not similarly facilitate the suppression of distractors.

In the present study, we asked participants about cue-distractor association learning at the end of each experiment. Surprisingly, in the informative and non-informative cue conditions, participants did not differ in their ability to recognise that the foil appeared more frequently at the cued location. This was supported by the exploratory qualitative analysis of responses to the open-ended question ‘Any other comments about what the cue might have been for?’, Only 11 participants (4.72% of those who provided a coded response) accurately identified the association. Several studies on statistical learning of distractor locations have used post-experiment assessments to test learning of the high frequency distractor location, where participants indicate where in the search array the distractor was most likely to appear (e.g. Wang & Theeuwes, 2018a, 2018b; Gao & Theeuwes, 2020). In these studies, participants performed at chance. Furthermore, when explicitly told the high-probability location suppression was not enhanced (Wang & Theeuwes, 2018b). These findings suggest that awareness of distractor locations does not develop in statistical learning experiments. However, contradictory findings have been reported using a broader range of distractor learning assessments (Vicente-Conesa et al., 2022). The high-frequency distractor location was identified by 58% of participants (Experiment 1), ranked highest in perceived frequency (Experiment 2) and estimated to have the highest frequency (although underestimating ~25% rather than 65%, Experiment 3). Similarly, Ferrante et al. (2023) found that 65% of participants correctly identified the high-probability distractor location, suggesting that conscious learning may be involved.

To the best of our knowledge, the current study is the first to directly assess cue-distractor learning following the use of explicit spatial cues. However, this should be considered a preliminary finding, as more systematic assessments (e.g. those used by Vicente-Conesa et al., 2022) would be more insightful. Nevertheless, our results suggest that participants did not learn the association between the cue and the foil. It is surprising that the relatively subtle manipulation of high-probability distractor locations has previously been successfully learnt by participants (Ferrante et al., 2023; Vicente-Conesa et al., 2022), whereas in the present study, the association with reliably informative, centrally located cues was not. One key factor may be that the distractor was a target feature matching foil rather than a singleton. It may be that the distractor must be salient for association learning to occur. Relatedly, we note that the presentation of the cue involved a colour change. Interestingly, the most common code in the exploratory qualitative analysis indicated that participants thought the cue itself was distracting, which supports attention capture by the informative cues in Experiments 1 and 3.

## Conclusion

In the present study, we attempted to replicate and extend the work of Munneke et al. (2008), who observed a distractor suppression enhanced by spatial cueing. We did not observe an effect in RT or in the interference time parameter from conflict diffusion models. The present study does not support the enhancement of attentional suppression through spatial cueing.

## Notes

1. Throughout this manuscript, we follow the recommended nomenclature for research on distraction recommended by Liesefeld et al. (2024).

2. As model fits to individual participant, data can sometimes be noisy with relatively low trial numbers. We also conducted a jackknife procedure on model parameters obtained by fitting each model to participant-averaged data (see Dambacher & Hübner, 2015). Results were consistent with the individual fitting reported here. See Supplemental Material S2 for details.

3. For the DTSP model: A/B = 0.145, C/D = 0.08, μ_t_ = 0.1, μ_fl_ = 0.07, μ_ss_ = 0.325, μ_rs2_ = 1.30 and *T*_er_ = 0.24. For the SSP: A/B = 0.05, *p* = 0.4, *r*_d_ = 0.05, *sd*_a_ = 1.5 *T*_er_ = 0.3.

4. Thanks to Dr. Heinrich Liesefeld for raising this point during peer review.
